# Red ginseng polysaccharide promotes ferroptosis in gastric cancer cells by inhibiting PI3K/Akt pathway through down-regulation of AQP3

**DOI:** 10.1080/15384047.2023.2284849

**Published:** 2023-12-05

**Authors:** Yan Wang, Wen-Xian Guan, Yuan Zhou, Xiao-Yu Zhang, Hai-Jian Zhao

**Affiliations:** a Department of General Surgery, Nanjing Drum Tower Hospital Clinical College of Traditional Chinese and Western Medicine, Nanjing University of Chinese Medicine, Nanjing, China; b Department of Gastrointestinal Surgery, The Affiliated Huai’an Hospital of Xuzhou Medical University, Huai’an, China

**Keywords:** Gastric cancer, red ginseng polysaccharide, aquaporin 3, ferroptosis, PI3K/Akt pathway

## Abstract

**Objective:**

This study aims to investigate the effect of red ginseng polysaccharide (RGP) on gastric cancer (GC) development and explore its mechanism.

**Methods:**

GC cell lines AGS were treated with varying concentrations of RGP (50, 100, and 200 μg/mL). AGS cells treated with 200 μg/mL RGP were transfected with aquaporin 3 (AQP3) overexpression vector. Cell proliferation, viability, and apoptosis were evaluated by MTT, colony formation assay, and flow cytometry, respectively. Real-time quantitative reverse transcription PCR (qRT-PCR) was used to detect the expression of AQP3. The levels of Fe2+, malondialdehyde, and lactate dehydrogenase were measured using their respective detection kits, and the reactive oxygen species levels was determined by probe 2’,7’-dichlorodihydrofluorescein diacetate. The expression of ferroptosis-related protein and PI3K/Akt pathway-related protein were assessed by western blot. In vivo experiments in nude mice were performed and the mice were divided into four groups (*n* = 5/group) which gavage administrated with 150 mg/kg normal saline, and 75, 150, 300 mg/kg RGP, respectively. Their tumor weight and volume were recorded.

**Results:**

RGP treatment effectively inhibited the proliferation and viability of AGS cells in a dosage-dependent manner and induced apoptosis. It induced ferroptosis in AGS cells, as well as inhibiting the expression of PI3K/Akt-related proteins. AQP3 overexpression could reversed the effect of RGP treatment on ferroptosis. Confirmatory in vivo experiments showed that RGP could reduce the growth of implanted tumor, with increased RGP concentration resulting in greater tumor inhibitory effects.

**Conclusion:**

RGP might have therapeutic potential against GC, effectively inhibiting the proliferation and viability of AGS cells.

## Introduction

Gastric cancer (GC) is a prevalent type of cancer worldwide, ranking fifth in morbidity and fourth in mortality among all cancers.^1^ The overall incidence of GC has decreased significantly in the past 5 y, largely due to the lower prevalence of *Helicobacter pylori* infections,^2^ but about one million new cases of GC are still diagnosed each year. Moreover, the incidence of gastroesophageal junction carcinoma is on the rise.^3^ Attributed to the lack of noticeable symptoms during the early stages of GC, most patients are diagnosed when the tumor has advanced, increasing the risk of tumor metastasis and drug resistance and leading to poor treatment outcomes.^4^ Despite significant progress in prolonging GC patient survival, over 70% of patients die from the disease, and the five-year survival rate remains between 20% and 30%.^5^ Therefore, there is an urgent need to discover new drugs or treatment protocols to enhance the treatment efficacy of patients with GC.

Aquaporins (AQPs), a family of hydrophobic integral membrane proteins belonging to the superfamily of special membrane integral proteins, are highly expressed in various cancers.^6^ AQP3 is an important member mainly expressed in upper gastrointestinal epithelial cells, and the regulatory role of AQP3 in cancer development has attracted increasing attention.^7^ For instance, studies have shown that AQP3 is differentially expressed in cancer tissues and peri-carcinomatous tissues of various organ systems and is involved in multiple signal transduction pathways in cancer cells.^8–10^ Jiang et al. found that AQP3 was highly expressed in GC cells and GC cell proliferation could be inhibited by interfering with AQP3 and decreasing autophagy levels.^11^ Another study revealed a strong connection between AQP3 and the PI3K/Akt pathway^12^ which plays an essential role in many normal cellular processes in various cell types. Moreover, recent studies have identified the PI3K/Akt pathway as a key signal involved in the induction of ferroptosis, a recently discovered oxidative non-apoptotic form of programmed cell death, in cancer cells.^13^ Ferroptosis occurs in mitochondria and is mainly triggered by iron-dependent lipid peroxidation, characterized by iron overload-induced accumulation of reactive oxygen species (ROS) and increased lipid peroxides.^14,15^ Programmed cell death is a hot topic in biomedical research, and inducing ferroptosis of cancer cells is a widely employed strategy in cancer therapy and has significant clinical value.

Chinese herbs and their derivatives have gained significant attention as accessible complementary medicines in the field of cancer therapy.^16^ Extensive investigations have been conducted to explore the effectiveness of Chinese herbal extracts in human cell lines, animal models, and clinical trials, revealing their remarkable efficacy, and minimal adverse effects in the treatment of diverse ailments, such as chronic diabetes, atherosclerosis, and various types of cancer.^17^ Red ginseng, a perennial herb classified under the Araliaceae family and the Panax genus, has been employed for medical purposes in Asia for countless millennia. In addition, red ginseng has been demonstrated efficacy in alleviating several ailments, such as inflammation, infection, fatigue, aging, and cancer.^18–20^ The active constituents of ginseng are ginsenosides, polyphenols, flavonoids, and polysaccharides,^21^ with ginsenosides as the most active one. Ginsenosides possess various pharmacological activities, while the pharmacological efficacy of ginseng polysaccharides have not been as actively investigated compared to ginsenosides. Red ginseng polysaccharide (RGP), one of the active components of red ginseng, has received much attention in cancer therapy.^22^ Shin et al. discovered that combining paclitaxel with RGP significantly increased macrophage activity and inhibited melanoma development in nude mice.^23^ In a recent study by Zhai et al., RGP was found to possess anticancer activities in lung and breast cancer cells by inducing cellular ferroptosis.^24^ Thus, it is believed that RGP may be effective in treating cancer.

Although there is no direct evidence to suggest the potential effect of RGP on AQPs in GC, the existing literature has reported the effects of RGP on ferroptosis and cancer, and the involvement of AQPs in multiple signal transduction pathways (including PI3K/Akt pathway) in cancer cells. Therefore, we hypothesized that RGP might have an indirect effect on AQPs by regulating the PI3K/Akt pathway or inducing ferroptosis in GC cells. The significance of this potential effect is that it could lead to the development of new drugs or treatment protocols for GC that target AQPs and the PI3K/Akt pathway or induce ferroptosis in cancer cells. Thus, as an attempt to shed more light on this topic, this present study was designed to explore the potential effects of RGP on GC development via *in vitro* and *in vivo* experiments and whether it could be considered a potential therapeutic agent for treating GC.

## Results

### Red ginseng polysaccharide inhibits proliferation and promotes apoptosis of AGS cells

**Figure 1.**
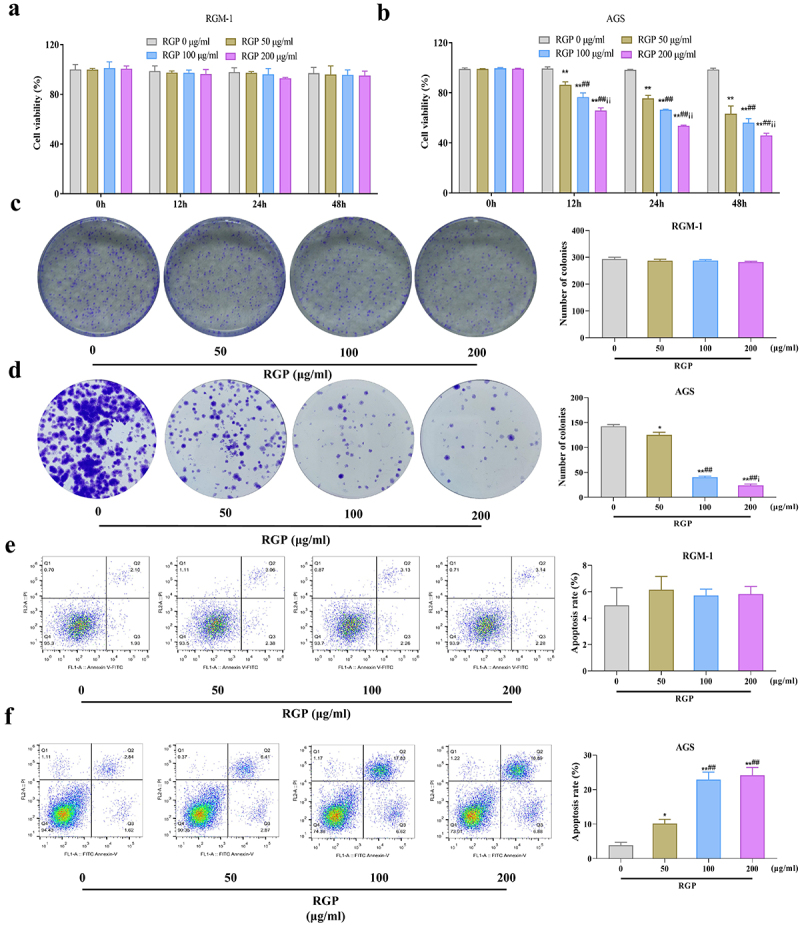
RGP inhibits proliferation and promotes apoptosis of AGS cells.

To investigate the potential therapeutic effects of RGP on GC and whether RGP would affect the normal gastric mucosa epithelial cells, we treated RGM-1 cells and AGS cells with different concentrations of RGP (50 μg/mL, 100 μg/mL, and 200 μg/mL) *in vitro*. The results showed that RGM-1 cells treated with different concentrations of RGP had no significant difference in cell proliferation (*p* > .05). However, AGS cells treated with RGP had significantly lower levels of proliferation compared to the control cells that received 0 μg/mL RGP (*p* < .01) in a dose-dependent manner, whereby increased RGP led to markedly reduced proliferation levels (Figure 1a, b). The colony formation assay indicated that RGM-1 cell viability was not affected after RGP treatment (*p* > .05) while AGS cell viability was significantly reduced after RGP treatment, also in a dose-dependent manner (Figure 1c, d). Furthermore, flow cytometry was performed to observe the effects of RGP on the apoptosis level of RGM-1 cells and AGS cells, and the results indicated that the apoptosis level of RGM-1 cells was not affected after RGP treatment (*p* > .05) while the apoptosis level of AGS cells increased with the increase of RGP concentration (Figure 1e, f). These results suggested that RGP significantly inhibited the proliferation and viability and promoted apoptosis of AGS cells.

(a – b) The effects of different concentrations of RGP on the proliferation level of RGM-1 cells (a) and AGS cells (b) by MTT assay. (c – d) The effects of different concentrations of RGP on the viability of RGM-1 cells (c) and AGS cells (d) by colony formation assay. (e – f) The effects of different concentrations of RGP on the apoptosis level of RGM-1 cells (e) and AGS cells (f) by flow cytometry.

Abbreviations: **p < 0.01, *vs*. RGP 0 μg/mL; ## *p* < 0.05, *vs*. RGP 50 μg/mL; ii *p* < 0.05, *vs*. RGP 100 μg/mL (*n* = 3). RGP, red ginseng polysaccharide.

### Red ginseng polysaccharide induces ferroptosis in AGS cells

**Figure 2.**
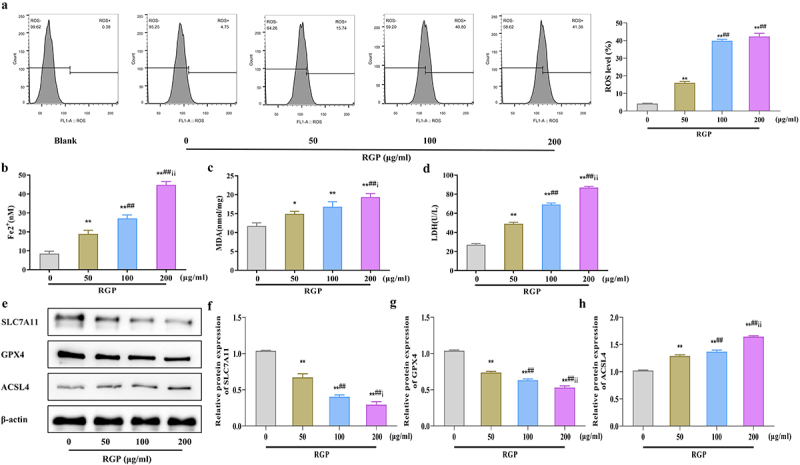
RGP induces ferroptosis in AGS cells.

Here, we explored the mechanism of action of RGP. First we detected ROS levels in AGS cells treated with RGP using a probe. The results showed that RGP significantly increased ROS accumulation in cells in a dosage-dependent manner, whereby increased RGP led to markedly increased cellular ROS accumulation (Figure 2a). Subsequently, we examined the levels of Fe^2+^ in cells and found that RGP could dose-dependently increase the accumulation of Fe^2+^ in cells (*p* < .01) (Figure 2b). Furthermore, RGP also markedly elevated the levels of malondialdehyde (MDA) and lactate dehydrogenase (LDH) in cells (*p* < .01) (Figure 2c, d). To further determine the relationship between RGP and ferroptosis, we detected the expression of ferroptosis-related proteins (solute carrier family 7 member 11 [SLC7A11], glutathione peroxidase 4 [GPX4], and acyl-CoA synthase long-chain family member 4 [ACSL4]) by western blot. The expression of SLC7A11 and GPX4 was significantly reduced (*p* < .01), while the expression of ACSL4 was gradually increased (*p* < .01) with increasing concentration of RGP (Figure 2e–h). Based on these findings, it could be speculated that RGP-induced ferroptosis in AGS cells.

(a) Flow cytometry results of the effect of different concentrations of RGP on the ROS levels in AGS cells. (b – d) Effects of different concentrations of RGP on (b) Fe^2+^, (c) MDA, and (d) LDH levels in AGS cells. (E – H) Western blot results of the effects of different concentrations of RGP on the expression of ferroptosis-related proteins (SLC7A11, GPX4, ACSL4) in AGS cells.

Abbreviations: *p < 0.05, **p < 0.01, *vs*. RGP 0 μg/mL; ## *p* < 0.01, *vs*. RGP 50 μg/mL; i *p* < 0.05, ii *p* < 0.01, *vs*. RGP 100 μg/mL (*n* = 3); RGP, red ginseng polysaccharide; ROS, reactive oxygen species; MDA, malondialdehyde; LDH, lactate dehydrogenase.

### Red ginseng polysaccharide inhibits the PI3K/Akt pathway in AGS cells

**Figure 3.**
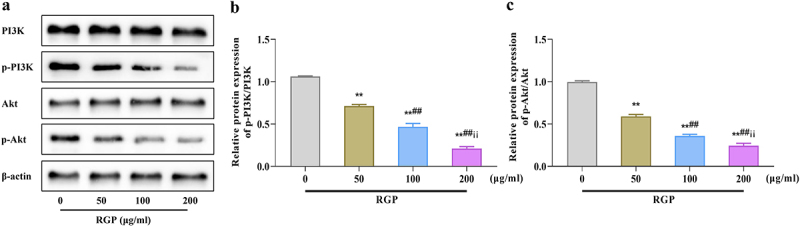
Inhibition of the PI3K/Akt pathway in AGS cells by RGP.

Next, we assessed the effects of RGP on the PI3K/Akt pathway by western blotting. The results showed that the levels of p-PI3K and p-Akt were significantly downregulated as the RGP concentration increased, and the ratios of p-PI3K/PI3K and p-Akt/Akt were also gradually decreased (*p* < .01) (Figure 3a–c). Collectively, RGP could induce ferroptosis in cells by inhibiting the activity of the PI3K/Akt pathway.

(a – c) Western blot results of the effects of different concentrations of RGP on the expression of the PI3K/Akt pathway-related proteins (PI3K, p-PI3K, Akt, p-Akt) in AGS cells.

Abbreviations: **p < 0.01, *vs*. RGP 0 μg/mL; ## *p* < 0.01, *vs*. RGP 50 μg/mL; ii *p* < 0.05, *vs*. RGP 100 μg/mL (*n* = 3); RGP, red ginseng polysaccharide.

### Red ginseng polysaccharide down-regulates the expression level of aquaporin 3 in AGS cells

**Figure 4.**
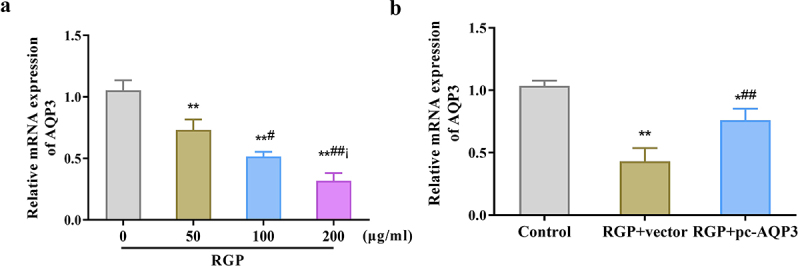
RGP down-regulates the expression level of AQP3 in AGS cells.

Here, we investigated the potential role of AQP3 in the mechanism of action of RGP in GC cells. The qRT-PCR results showed that RGP decreased AQP3 expression in AGS cells in a dose-dependent manner (*p* < .01) (Figure 4a), suggesting that AQP3 may play a role in the inhibitory effect of RGP on GC cells. To further explore this potential role, AQP3 was overexpressed in cells treated with RGP. The results showed that in cells treated with RGP at a concentration of 200 μg/mL, transfection of pcDNA3.1-AQP3 vector led to an upregulation of AQP3 expression, and this overexpression reversed the inhibitory effect of RGP on AQP3 expression (*p* < .01) (Figure 4b). These results indicated that the inhibitory effect of RGP on GC cells might be mediated, at least in part, by the downregulation of AQP3 expression.

(a) qRT-PCR results of the effect of different concentrations of RGP on AQP3 expression in AGS cells, **p < 0.01, *vs*. RGP 0 μg/mL; #*p* < 0.05, ##*p* < 0.01, *vs*. RGP 50 μg/mL; i *p* < 0.05, *vs*. RGP 100 μg/mL. (b) qRT-PCR results of the expression level of AQP3 in AGS after transfection with AQP3 overexpression plasmid, *p < 0.05, **p < 0.01, *vs*. Control; ##*p* < 0.01, *vs*. RGP + vector (*n* = 3).

Abbreviations: RGP, red ginseng polysaccharide; AQP3, aquaporin 3; qRT-PCR, real-time quantitative reverse transcription PCR.

### Red ginseng polysaccharide increases ferroptosis in AGS cells by downregulating aquaporin 3

**Figure 5.**
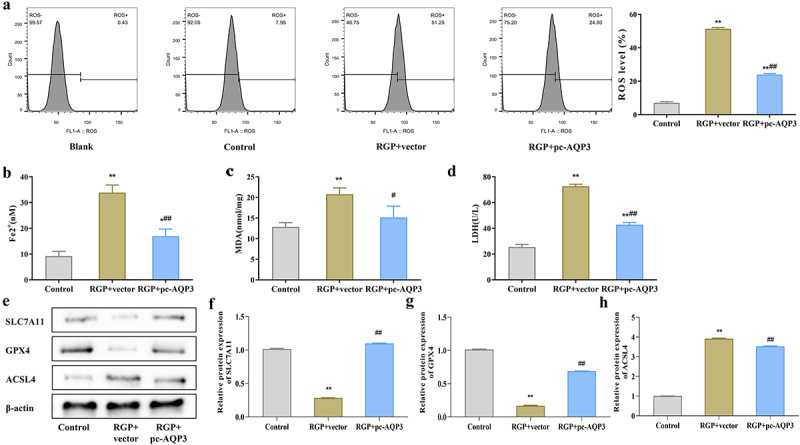
RGP increases ferroptosis in AGS cells by downregulating AQP3.

After overexpressing AQP3, we investigated whether RGP-induced ferroptosis in AGS cells through AQP3. Ferroptosis-related indicators was observed in AGS cells treated with RGP and transfected with an AQP3 overexpression plasmid. The results showed that upregulating the expression of AQP3 in RGP-treated cells (RGP + pc-AQP3) significantly reduced the level of lipid ROS (Figure 5a), and the levels of Fe^2+^, MDA and LDH were also decreased compared to the RGP + vector group (*p* < .05) (Figure 5b–d). Western blot analysis showed that the protein expression levels of SLC7A11 and GPX4 were significantly elevated (*p* < .01), while that of ACSL4 was significantly reduced (*p* < .01) in the RGP + pc-AQP3 group compared to the RGP + vector group (Figure 5e–h). Therefore, these findings suggested that increasing AQP3 expression level could reverse the effect of RGP on inducing ferroptosis in AGS cells.

(a) ROS levels in AGS cells of different treatment groups by flow cytometry. (b) Detection of (b) Fe^2+^, (c) MDA, and (d) LDH levels in cells of different treatment groups. (e-h) Expression of ferroptosis-related proteins (SLC7A11, GPX4, and ACSL4) in cells of different treatment groups by western blot.

Abbreviations: *p < 0.05, **p < 0.01, *vs*. Control; #*p* < 0.05, ##*p*<0.01, *vs*. RGP + vector (*n* = 3); RGP, red ginseng polysaccharide; ROS, reactive oxygen species; MDA, malondialdehyde; LDH, lactate dehydrogenase.

### Red ginseng polysaccharide inhibits the PI3K/Akt pathway in AGS cells by downregulating aquaporin 3

**Figure 6.**
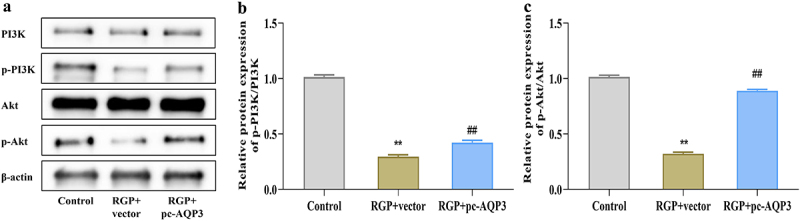
RGP inhibits PI3K/Akt pathway in AGS cells by downregulating AQP3.

To understand the relationship between AQP3 overexpression and PI3K/Akt pathway activity in RGP-treated AGS cells, we investigated whether the overexpression of AQP3 could reverse the inhibitory effect of RGP on PI3K/Akt pathway activity. Western blot experiments revealed that the RGP + pc-AQP3 group had significantly higher levels of p-PI3K and p-Akt, as well as the ratios of p-PI3K/PI3K and p-Akt/Akt, compared to the RGP + vector group (Figure 6a–c), thereby indicating that RGP might downregulate AQP3 to affect PI3K/Akt pathway activity.

(a – c) Western blot results of the expression of PI3K/Akt pathway (PI3K, p-PI3K, Akt, p-Akt)-related proteins from different treatment groups in AGS cells.

Abbreviations: **p < 0.01, *vs*. Control; ##*p* < 0.01, *vs*. RGP + vector (*n* = 3). RGP, red ginseng polysaccharide; AQP3, aquaporin 3.

### *In vivo* confirmation of the potential efficacy of red ginseng polysaccharide

**Figure 7.**
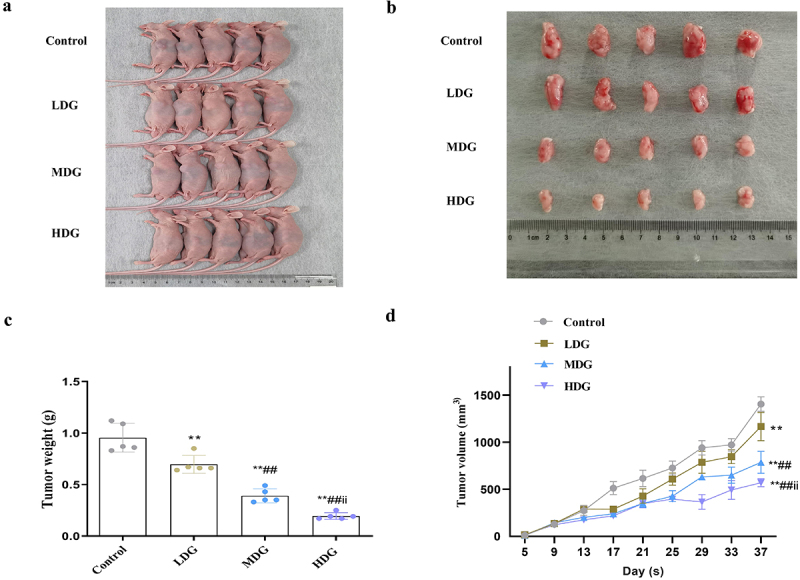
*In vivo* experiments indicate the potential therapeutic significance of RGP in inhibiting the growth of AGS cells.

Lastly, confirmatory experiments were performed in nude mice to determine whether these *in vitro* experiment results could be translated into living organisms. *In vivo* subcutaneous tumor implantation experiments indicated that RGP could effectively reduce the growth of the implanted tumors (Figure 7a–d). In addition, increased concentration of RGP led to significantly greater inhibitory effects on tumor weight and volume (Figure 7a–d). Taken together, *in vivo* experimental findings provided some preliminary insights into the potential value of RGP in the treatment of GC.

(a) Gross examination of nude mouse subcutaneous tumor models. (b – d) The excised tumoral tissues showing a significant decrease in (b) size, (c) weight, and (d) volume with increasing RGP concentration.

Abbreviations: **p < 0.01, *vs*. Control group; ##*p* < 0.05, *vs*. LDG group; ii *p* < 0.05, *vs*. MDG group (*n* = 5). RGP, red ginseng polysaccharide. LDG group, 75 mg/kg RGP; MDG group, 150 mg/kg RGP; HDG group, 300 mg/kg RGP.

## Discussion

GC is often diagnosed at an advanced stage, making its treatment outcomes (except for early-stage GC) remain unsatisfactory.^25^ Chinese herbs have shown promise in fighting against GC, with RGP being one of the active components of red ginseng that offers potential for cancer treatment.^16,18–20^ Truong et al. demonstrated the significant anti-tumor efficacy of RGP in nude mice transplanted with human colon adenocarcinoma cells.^26^ RGP regulated the tumor immunosuppressive microenvironment by enhancing the tumoricidal activity of peritoneal macrophages against B16 melanoma cells, as described by Choi et al..^27^ In this study, we showed that RGP could inhibit the proliferation and viability of GC cells and promote apoptosis in a dose-dependent manner, but not affect the proliferation, viability and apoptosis of normal gastric mucosa epithelial cells RGM-1. We further proved that RGP might exert its effects on GC cells through regulating AQP3 and PI3K/Akt signaling activity. Importantly, this is the first study to demonstrate the inhibitory effect of RGP on AGS cell activity *in vitro* and *in vivo*. The aberrant expression of AQP3, a key molecule in cancer therapy, has been reported to be associated with various cancer-related processes, including cell proliferation, invasion, migration, and apoptosis, in various cancers. Zhu et al. previously demonstrated that AQP3 promoted GC cell proliferation by increasing intracellular lipid metabolism and improving dysregulation.^28^ Furthermore, the observation that higher concentrations of RGP led to greater tumor inhibitory effects may be useful for guiding dosing strategies in future clinical trials. Overall, the study provides promising evidence for the potential clinical use of RGP in treating GC and warrants further investigation.

Systemic therapy, especially radiotherapy, has shown beneficial efficacy in prolonging the survival of GC patients. However, these therapies are often associated with various adverse events, including failure of the hematopoietic system and severe immunosuppression.^29^ In this regard, RGP has been proposed as a candidate with promising radioprotective effects. It was reported to significantly increase hematopoietic and immune cells, such as bone marrow cells and spleen cells, in irradiated mice.^30^ In addition, Kim et al. observed that an acidic polysaccharide of *Panax ginseng* increased bone marrow cell viability *in vitro* and *in vivo*, which was not suppressed by gamma radiation, highlighting the potential of polysaccharide of *Panax ginseng* as a radioprotective agent, at least for prophylactic purposes.^29^ Thus, a potential application can be the development of RGP-based therapies, which can be administered either alone or in combination with other treatments (such as chemotherapy or radiation therapy) to enhance their effectiveness. In addition, the aforementioned findings can also inform the development of diagnostic and prognostic tools that help identify patients who are most likely to benefit from RGP-based therapies and monitor their response to treatment.

Ferroptosis, a recently discovered type of cell death, has shown great potential in cancer therapy.^31^ Ferroptosis is involved in numerous physiological and pathological processes of various cancers.^32^ Specific lipid peroxides, such as ROS and MDA, are generally considered as markers of ferroptosis. Moreover, GPX4, NADPH oxidase, SLC7A11, and ACSL4 indirectly regulate iron metabolism and lipid peroxidation metabolic processes, thereby promoting ferroptosis.^33^ Ferroptosis has been found to be associated with various gastrointestinal cancers, such as GC, hepatocellular carcinoma, and pancreatic cancer.^34^ In our study, RGP significantly increased the accumulation of ROS, MDA, LDH, and Fe^2+^, decreased the protein expression of SLC7A11 and GPX4, and upregulated the protein level of ACSL4 in AGS cells, indicating that RGP can induce ferroptosis in AGS cells. However, the effect of RGP on inducing ferroptosis in AGS cells was greatly weakened after overexpressing AQP3, suggesting that RGP may induce ferroptosis in AGS cells by inhibiting AQP3 expression.

In addition to its role in lung inflammation, cell survival, and oxidative stress, the PI3K/Akt signaling pathway is also closely related to ferroptosis. Specifically, PI3K/Akt signaling promoted the translation of GPX4 protein by inhibiting the downstream molecule 4E-BP1, thereby suppressing ferroptosis.^35^ Yi et al. showed that PI3K/Akt also inhibited ferroptosis through the SREBP1-SCD-1 axis.^36^ Hayes et al. discovered that PI3K/Akt increased the stability of Nrf2 to affect ferroptosis by inhibiting glycogen synthase kinase-3β.^37^ Collectively, the activity of the PI3K/Akt pathway can regulate ferroptosis in cells. Importantly, previous studies have demonstrated a close relationship between the expression level of AQPs and PI3K/Akt signaling pathway activity. For example, Cao et al. reported the significance of FGFR-PI3K and FGFR-ERK signaling in AQP3 expression in breast cancer.^38^ Zhang et al. also suggested that AQPs regulated PI3K/AKT pathway activity in the expression and regulation of placenta and fetal membranes.^39^ In our study, we found that RGP could significantly inhibit the activation of the PI3K/Akt pathway, while the inhibitory effect of RGP on the PI3K/Akt pathway was significantly limited after overexpression of AQP3. Therefore, it is evident that RGP functions by affecting PI3K/Akt signaling activity through AQP3.

A multifaceted approach is necessary to further elucidate the efficacy of RGP and the potential of AQP3 in GC treatment and prepare for clinical trials. This approach should encompass extensive *in vitro* and *in vivo* studies using various GC cell lines and animal models, such as mice with xenografted gastric tumors, so as to validate the role of AQP3 and RGP-induced ferroptosis within a complex biological context. Concurrently, a thorough investigation of the molecular mechanisms underlying RGP-induced ferroptosis and AQP3’s involvement, including the relationship of RGP with other proteins and signaling pathways associated with ferroptosis, is vital in addition to the PI3K/Akt pathway, especially considering the heterogeneous nature of GC. Identifying potential biomarkers that predict RGP treatment efficacy and AQP3-targeted therapy success will also help develop personalized treatment strategies. Additionally, examining the synergistic effects of combining RGP treatment with other anticancer agents, such as chemotherapy, targeted therapy, or immunotherapy, can show promising insights into novel strategies for enhancing overall treatment efficacy and circumventing possible resistance to RGP-induced ferroptosis. Further, a thorough safety and toxicity assessment in preclinical models will be crucial to understanding the potential risks and benefits of these treatments before consideration for clinical trials. Upon gathering sufficient preclinical data, phase I clinical trials can evaluate the safety, tolerability, and optimal dosing of RGP and/or AQP3-targeted therapy, followed by phase II and III trials to compare their efficacy, safety and overall survival benefits to standard therapies. Lastly, devising patient stratification strategies based on AQP3 expression levels, genetic background, and other pertinent factors will ensure that clinical trials are tailored to target patient populations most likely to benefit from the treatments. By pursuing these steps, researchers may gain a deeper understanding of the actual potential of RGP efficacy and AQP3’s promise in GC management while laying the groundwork for subsequent clinical trials.

## Conclusion

To summarize, our findings suggest that RGP has anti-tumor effects *in vivo* and *in vitro*. RGP inhibits PI3K/Akt signaling activity by downregulating AQP3 expression, promotes ferroptosis in AGS cells, reduces proliferation and viability, and ultimately induces apoptosis. RGP demonstrated great prospects as a drug for consideration in GC treatment. However, translating this experimental findings to clinical practice in GC patients requires further research, including preclinical studies and clinical trials, to establish the safety and efficacy of RGP-based therapies. If successful, these therapies can offer a promising new option for treating GC, a disease currently with limited treatment options and poor prognosis.

## Materials and methods

### Cell culture and treatment

The normal gastric mucosa epithelial cells RGM-1 and the GC cell line AGS were cultured in RPMI-1640 medium (Gibco, Carlsbad, CA, USA) supplemented with 10% fetal bovine serum and 1% double antibody and incubated in a humidified incubator at 37°C with 5% CO_2_. To assess the effect of RGP, AGS cells were treated with RGP at concentrations of 0 μg/mL, 50 μg/mL, 100 μg/mL, and 200 μg/mL. To investigate the relationship between AQP3 and RGP, AGS cells treated with 200 μg/mL RGP were transfected with an AQP3 overexpression plasmid (pcDNA3.1-AQP3) (RGP + pc-AQP3 group) or blank plasmid (RGP + vector group) using lipo2000 (Invitrogen, CA, USA). After 24 hours, the relevant indicators of the cells were measured.

### MTT assay

AGS cells were seeded into 96-well plates at a density of 1.0 × 10^3^ cells/well and treated with different concentrations of RGP for 0 h, 12 h, 24 h, and 48 h. After the treatment, the cells were assessed for their proliferation ability using the MTT assay (Trevigen, MC, USA). Briefly, MTT solution (5 mg/L) was added to each well, and the cells were incubated for an additional 4 h. The medium containing the MTT solution was then carefully removed, and 100 μL of DMSO was added to each well. After 15 min, the relative absorbance at 570 nm was measured using a microplate spectrophotometer (Agilent Technologies, Cernusco sul Naviglio [MI], Italy).

### Colony formation assay

The treated cells were seeded in 6-well plates at a density of 4 × 10^2^ cells/well and incubated at 37°C with 5% CO_2_ for 10 d to form colonies. Following the completion of cloning, the cells were washed twice with phosphate-buffered saline (PBS) and fixed with 4% paraformaldehyde (Beyotime, Shanghai, China) for 20 min. Washed again with PBS, the cells were stained with 5% crystal violet (Beyotime, Shanghai, China) for 20 min. The number of colonies was counted under a microscope.

### Flow cytometry

AGS cells were double-stained with Annex V-FITC/PI to observe apoptosis using the FITC Annex V Apoptosis Detection Kit (vazyme, Nanjing, China). The cells were resuspended in binding buffer at a concentration of 1 × 10^6^ cells/mL after treatment with 0.05% trypsin in PBS with 0.02% EDTA and three washes with PBS. Next, 100 μL of the resulting solution was transferred to culture tubes, and 5 μL of FITC Annex V and 5 μL of PI were added. The mixture was incubated in the dark for 15 min, after which 400 μL of binding buffer was added to each tube for analysis by flow cytometry (Becton Dickinson, San Diego, CA, USA).

### Detection of reactive oxygen species

To observe the production of ROS, the cells were stained with the probe 2’,7’-dichlorodihydrofluorescein diacetate (DCFH-DA) (Beyotime, Shanghai, China). Then the stained cells were collected into centrifuge tubes and incubated with DCFH-DA for 30 min in the dark. The samples were analyzed by flow cytometer, and the fluorescence at an excitation/emission wavelength of 488/525 nm was measured.

### Detection of Fe2 +, malondialdehyde, and lactate dehydrogenase levels

The levels of Fe^2+^, malondialdehyde (MDA), and lactate dehydrogenase (LDH) in cells were measured using commercial kits from Nanjing Jiancheng Bioengineering Institutes (Nanjing, China). After being transferred into a centrifuge tube, AGS cells were fully lysed using an ultrasonic disruptor for 5 min (40 W of power, 2 s of work time, 3 s of interval). After centrifugation at 12,000 rpm for 10 min at 4°C, the supernatant was transferred to a new centrifuge tube. Then the levels of Fe^2+^, MDA, and LDH in the cells were detected using the corresponding kits.

### Western blot

AGS cells were seeded in 6-well plates at a density of 1 × 10^6^ cells/well and washed twice with PBS after the corresponding treatment. Total protein was extracted by lysing the cells with RIPA lysis solution (BioSharp, Shanghai, China). The concentration of the extracted protein was determined using a BCA kit (Thermo Fisher Scientific, Rockford, USA). A total of 20 μg of protein was boiled and denatured in 5 × loading buffer and then separated by SDS-PAGE. The protein was transferred to a PVDF membrane and blocked with 5% nonfat dry milk for 2 h. Primary antibodies (Abcam, Cambridge, UK) against SLC7A11, GPX4, ACSL4, PI3K, p-PI3K, p-Akt, and p-Akt were added for overnight incubation at 4°C on an incubator shaker. The membrane was then washed three times with TBST before adding secondary antibodies, which were incubated at room temperature for 1 h, followed by three rinses with TBS. ECL chemiluminescence reagent was added to the membrane, which was then placed in a gel imaging system (Alpha, CA, USA) to develop and collect images. The gray level of the protein bands was determined using Image J software, and the relative protein expression was calculated with β-actin as an internal control.

### Real-time quantitative reverse transcription PCR

Total RNA was extracted from cells using TRIzol reagent (Invitrogen, CA, USA) according to the manufacturer’s instructions. The total RNA was then reverse transcribed into cDNA using M-MLV reverse transcriptase (Promega, Milano, Italy). The expression level of mRNA was detected using the SYBR GREEN kit (TaKaRa, Japan) following the kit instructions. The qPCR reaction mixture contained 1 µg of RNA, 10 µL of the reaction solution, 1 µL of RT primers, and 1 µL of dNTPs, with a final volume of 15 µL. The mixture was heated at 70°C for 5 min and quickly cooled on ice. RT buffer (4 µL), M-MLV reverse transcriptase (1 µL), and RNase inhibitor (1 µL) were then added to the mixture on ice. RT reactions were performed at 42°C for 60 min and at 85°C for 5 min. Using GAPDH as an internal control, the experimental data obtained by qRT-PCR were used to calculate the relative expression of the target gene using the 2^−ΔΔCt^ method. The primer sequences are shown in Table 1.

**Table 1.** Quantitative primers.

RNA	Sequences (5’ to 3’)
AQP3	F: 5’- CCGTGACCTTTGCCATGTGCTT
	R: 5’- TTGTCGGCGAAGTGCCAGATTG
GAPDH	F: 5’- GTCTCCTCTGACTTCAACAGCG
	R: 5’- ACCACCCTGTTGCTGTAGCCAA

### Subcutaneous tumor formation in nude mice

Nude mice are a suitable model for tumor growth because they lack thymus and have T-cell immunodeficiency. Herein, we aimed to investigate tumor formation in nude mice using the gastric cancer cell line AGS and explore the effects of red ginseng on the subcutaneous tumor growth. For this experiment, 6–8 week-old BALB/c nude mice (Jiangsu Jicuiyaokang Biotechnology Co., Ltd., Jiangsu, China) were used and divided into four groups (five mice per group) which gavage administrated with 150 mg/kg normal saline (Control group), 75 mg/kg RGP (LDG group), 150 mg/kg RGP (MDG group), and 300 mg/kg RGP (HDG group), respectively. All animal experiments were approved by the Institutional Animal Care and Use Committee (IACUC) and conducted in accordance with the guidelines for the care and use of laboratory animals.

Then, a cell suspension of 0.3 ml cultured AGS cells (DMEM medium supplemented with 10% fetal bovine serum at standard conditions, adjusted to a concentration of 6 × 10^5^/ml in PBS after centrifugation) was injected subcutaneously into the back of each mouse using a 1 ml syringe after they were sterilized with 75% medical alcohol. The injection site was gently pressed with a cotton swab to prevent leakage, and tumor growth was observed after 7 d of injection in each group. All animals were kept in a specific pathogen-free environment with standard housing conditions.

The subcutaneous tumor size was measured every 4 d using a caliper, and the tumor volume was calculated using the formula: Volume = (Length × Width^2^/2. The tumor weight was measured on an electronic balance (Shenyang Longteng Electronics 053385) after the mice were sacrificed at the end of the experiment.

### Statistical analysis

All experiments were repeated at least three times, and the data are presented as mean ± standard deviation. Statistical analyses were performed using GraphPad Prism software (version 7; GraphPad Software, Inc.). A non-independent samples t-test was used to assess the differences between two groups, and one-way analysis of variance followed by Tukey’s post hoc test was used to compare multiple groups. A *p*-value less than 0.05 was considered statistically significant.

## Data Availability

Data used to support the findings of this study are available from the corresponding author upon request.
